# Surgical treatment of primary cardiac valve tumor: early and late results in eight patients

**DOI:** 10.1186/s13019-016-0406-2

**Published:** 2016-02-19

**Authors:** Yong Wang, Xuefeng Wang, Yingbin Xiao

**Affiliations:** Institute of Cardiovascular Surgery, Xinqiao Hospital, Third Military Medical University, No. 183 Xinqiao Rd, Shapingba, Chongqing 400037 China

**Keywords:** Primary cardiac valve tumor, Valvuloplasty, Valve replacement

## Abstract

**Background:**

To report early and late outcomes of patients with the primary cardiac valve tumor undergoing surgical treatment over a 30-year period in our cardiovascular center.

**Methods:**

From January 1980 to December 2014, a total of 211 patients with primary cardiac tumors accepted surgical treatments, of which only 8 (3.8 %) were primary cardiac valve tumor patients in our surgical center of cardiovascular.

**Results:**

The diagnosis was identified by echocardiography preoperatively and pathological analysis postoperatively. All patients underwent intracardiac procedures with extracorporeal circulation. Intracardiac procedures included resection of tumor on leaflet in 2 patients (25 %), resection of tumor and native valvuloplasty in 2 patients (25 %), resection of neoplasm and replacement of native valve with prosthetic valve in 4 patients (50 %). One man was performed a resection of tumor on aortic noncoronary leaflet and a coronary artery bypass graft. Eight cases of primary valve tumor occured in all of four cardiac valves. The majority of valvular tumor was myxoma in 3 cases (37.5 %), followed by the papillary fibroelastomas in 2 cases (25 %). There were one rhabdomyoma (12.5 %), one lipoma (12.5 %) and one mild malignant sarcoma (12.5 %). The mitral valve was the most commonly original valve (62.5 %). There was pulmonic (12.5 %), aortic (12.5 %) and tricuspid (12.5 %) valve tumor each one patient. There was no death and recrudescence in the series. Follow-up of all patients ranged from 1 to 16 years (mean 7.06±4.24 years). There was no recrudesce and cardiac valve dysfunction.

**Conclusion:**

The incidence of primary valve tumor was very low. More understanding of the rare disease and widespread use of echocardiography would greatly improve the diagnosis of primary valve tumor in the early stage. Echocardiography could detect millimeters in diameter neoplasms on cardiac valve. The diagnoses were based on imaging findings and the classical triad symptoms associated with the hemodynamic abnormalities, the organ embolism and the systemic symptoms directly from tumors. The intraoperative frozen sections and postoperative pathology analysis provided accurate diagnosis and supported the treatment strategies. Early diagnosis and intervention were keys to reserve the normal original valve function. Prompt surgical resection is necessary to prevent potential critical events.

## Background

Primary cardiac tumors are rare, and primary cardiac valve tumors are extremely rare, the reported incidence of primary cardiac valve tumors was less than 10 % of all primary cardiac tumors [1–3]. From January 1980 to December 2014, a total of 211 patients with primary cardiac tumors accepted surgical treatments, of which only 8 (3.8 %) were primary cardiac valve tumors in the department of cardiovascular surgery of our hospital. We reported 8 cases of primary cardiac valve tumors and presented a comprehensive review of literatures.

## Patients and methods

This study was approved by the ethics committee of our hospital. All of patients we studied were pure Han Chinese descents. Data was obtained from our hospital medical database. Follow-ups were obtained by letters or calls to assess functional status, and results of late echocardiography and laboratory examinations were reviewed. There were 6 male (75 %) and 2 female (25 %) patients, aged 33.00 ± 23.02 (1–63) years. Body weight was 51.65 ± 22.47 (8–76) kg.

One patient (12.5 %) was asymptomatic, 6 patients (75 %) manifested shortness of breath, fatigue and weakness, 2 patients (25 %) presented with vertigo or dizziness, 2 patients (25 %) had paroxysmal chest pain. Hemiplegia and blurring of vision caused by cerebral infarction were in 2 patients (25 %). The symptomatic patients had a disease course of 7.00 ± 7.78 (1–25) months.

Chest X-ray showed cardiac shadow enlargement in 6 cases (75 %). EEG indicated atrial fibrillation rhythm in 3 cases (37.5 %), left ventricular hypertrophy in 4 cases (50 %), ST-T wave changed in 3 cases (37.5 %). Focal cerebral infarctions were confirmed by CT scan in 2 cases (25 %). The Coronary heart disease was diagnosed by angiography in 1 case (12.5 %). Masses on valve were detected through preoperative echocardiography in all patients. All patients had a good left ventricular function with an ejection fraction of 64.87 ± 6.10 (55–74) percent. Blood bacterial cultures were negative. There were 3 patients (37.5 %) with mild to moderate anemia. One patient (12.5 %) with a giant pulmonary valve tumor was hypohepatia due to obstruction of the right ventricular outflow track.

**Fig. 1:**
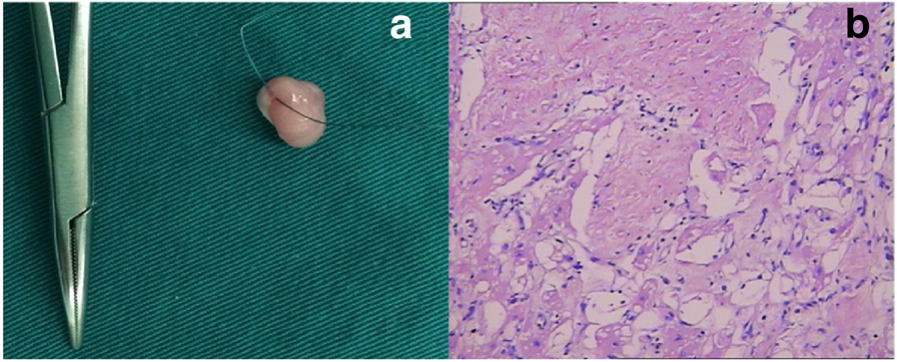
(Case 4, Mitral valve rhabdomyoma) **a**: A light-red elliptical neoplasm with smooth surface, shaved completely from the mitral anterior leaflet, 1 × 0.8 × 0.4 cm^3^. **b**: Histologically, the tumor was highly cellular and composed of somewhat pleomorphic, polygonal muscle cells admixed with spindle-shaped cells. There was “spider web” appearance in some tumor cells which has been known as the classic microscopic finding for rhabdomyoma. The tumor showed widely myxoid degeneration. (Hematoxylin and eosin)

**Fig. 2:**
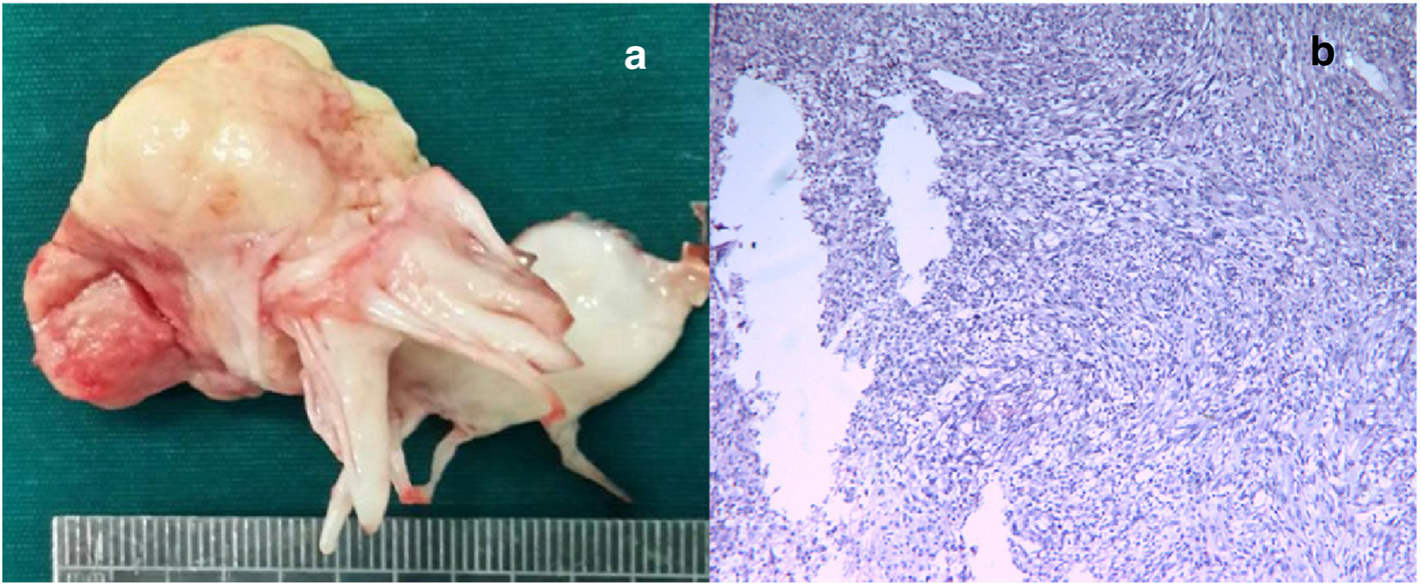
(Case 5, Malignant mitral valve tumor) **a**: A Yellow neoplasm looked like cauliflower with granular surface, destructively growth from the posterior leaflet of MV, 4.8 × 4.0 × 2.5 cm^3^. **b** Histology of mesenchymal sarcoma. Tumor cells of different sizes and shapes have pleomorphic nuclei and much secretion of fluid matrix. Karyokinesis is visible

**Fig. 3:**
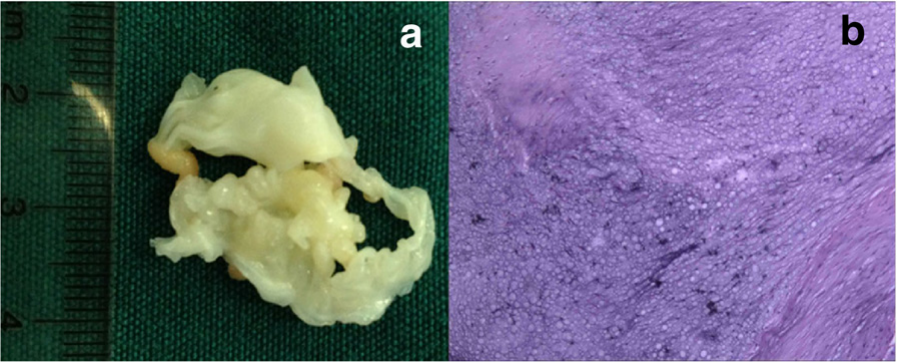
(Case 7, Mitral valve multiple myxoma) **a**: There are more than 10 pieces of neoplasm on leaflets of mitral valve. White neoplasms looked like granular surface. **b** Histologic aspect of the tumor, with the Characteristic Acid-Mucopolysaccharide Matrix and Embedded Polygonal Cells. (Hematoxylin and eosin)

**Fig. 4:**
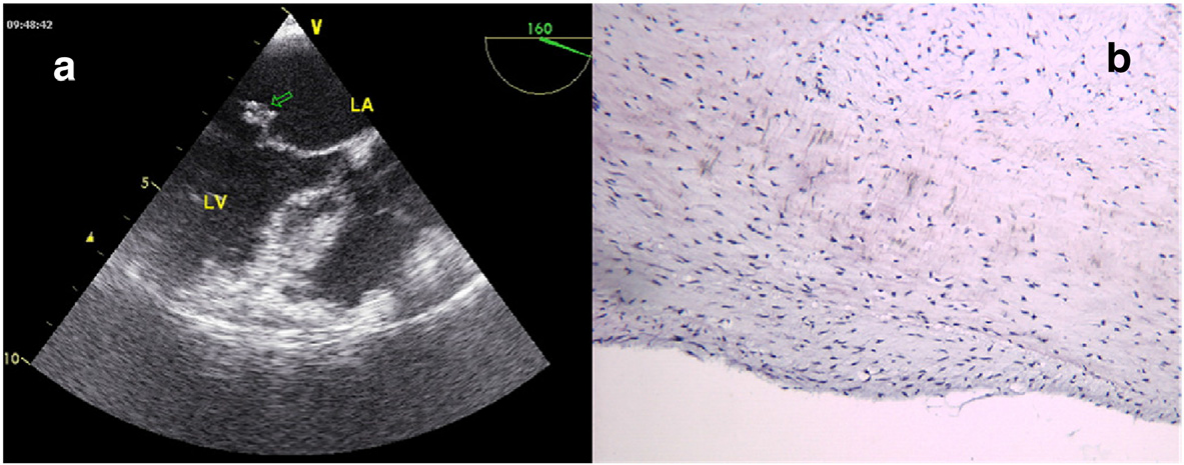
(Case 6, Mitral valve papillary fibroelastoma) **a**: The echocardiogram shows a 1.2 × 1.0 cm^2^ mass attached to the anterior leaflet of mitral valve. The arrow indicates the neoplasm on the atrial side of mitral valve. **b** The low power photomicrograph showing fibroustissue hyaline degeneration papillary hyperplasia and mucinous degeneration. (Hematoxylin and eosin)

**Fig. 5:**
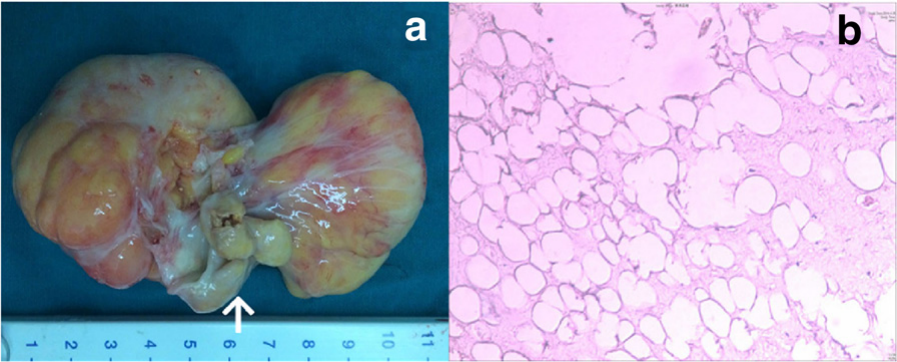
(Case 8, Giant pulmonary valve lipoma.) **a**: A giant smooth surface neoplasm growed from the pulmonary valve, 10 × 5.5 × 4.5 cm^3^. The arrow indicates the original pulmonary valve. **b** Histologic aspect of the tumor. The tumor consists of well-circumscribed lobulated adipose tissue and uniform mature fat cells. (Hematoxylin and eosin)

The eight patients were performed with intravenous combined anesthesia, endotracheal intubation, a median sternotomy, and general establishment of extracorporeal circulation with moderate hypothermia (nasopharyngeal temperature 26 to 30 °C). The myocardial protection was provided by delivering intermittent antegrade cold blood cardioplegia. Time of cardiopulmonary bypass was 74.38 ± 20.74 (45–102) minutes; the cross-clamping aorta was 56.13 ± 18.87 (32–86) minutes. Case 1 and 3 were performed excision of the tumor on the anterior mitral leaflet and on the tricuspid septal leaflet respectively. Case 2 with a neoplasm on the aortic noncoronary leaflet and a coronary artery disease was performed excision the tumor and coronary artery bypass grafting for the left anterior descending artery at the same time. Patient 4 (Fig. 1), a 1-year-old boy, underwent an excision of a neoplasm on anterior commissure of mitral valve. Patient 5 (Fig. 2) and 7 (Fig. 3) were both performed excision of tumor and native mitral leaflet, replaced with a mechanical mitral valve. Patient 6 (Fig. 4), a 26-year-old lady who wanted to breed, underwent an excision of a tumor on the mitral valve and replacement with a bioprothetic valve. Patient 8 (Fig. 5) with a giant tumor on the right ventricular outflow tract was performed excision of neoplasm and destroyed native pulmonary valve, replaced with a bioprothetic valve. Intraoperative transesophageal echocardiography confirmed without original valvular regurgitation and prosthetic valve dysfunction. We used intraoperative frozen sections (4/8 cases, 50 %) and neoplasm appearance to decide our surgical strategies. Pathologic analysis confirmed the accurate diagnosis. The only malignant patient (Fig. 2), case 5, had underwent intravenous systemic chemotherapy for three cycles within 6 months postoperatively. The detailed clinical data and pathological findings are showed in Table 1.

**Table 1:** clinical data of 7 cases of primary cardiac valve tumor

Case	Age (years)	Sex	Course (months)	Location	Measuring (cm^3^)	Gross Inspection	Histological Results	Surgical Procedures	Follow-up (years)
1	21	M	1	MV (anterior leaflet)	1.5 × 0.8 × 0.5	Light-yellow, gelatinous, relatively loose neoplasm	Myxoma	Resection of neoplasm and its original site in leaflet, cauterization and the leaflet repair	8 Normal MV function, NYHAI
2	63	M	25	AV (noncoronary leaflet)	1.5 × 1.1 × 0.8	White elliptical neoplasm with looking like fronds surface	Papillary fibroelastoma	Complete resection of neoplasm, cauterization and CABG	7 Mild AV regurgitation, NYHAII
3	33	M	4	TV (septal leaflet)	2.0 × 1.5 × 0.7	Light-yellow, gelatinous, relatively loose neoplasm	Myxoma	Complete resection of neoplasm and cauterization and the leaflet repair	7.5 Normal TV function, NYHA I
4	1	M	5	MV (anterior leaflet)	1.0 × 0.8 × 0.4	Light-red elliptical neoplasm with smooth surface Fig. 1a	Rhabdomyoma desmin (++)、vimentin (++)、Ki-67 (1–2 %+)、Masson (+) Fig. 1b	Complete resection of neoplasm and cauterization	16 Normal MV function, NYHAI
5	48	F	3	MV (posterior leaflet)	4.8 × 4.0 × 2.5	Yellow neoplasm looked like cauliflower with granular surface, destructively growth from the posterior leaflet of MV Fig. 2a	Mesenchymal sarcoma (SMA+, Vimentin+,CD34+,CD32+,MyoD1+,CD117+) Fig. 2b	Complete resection of neoplasm and mitral valve,subvalvular apparatus, replacement with 27-mm SJM mechanical valve	6 Normal mechanical valve function, Without anticoagulation comlications INR 2.0 ~ 2.5, NYHAII
6	26	F	6	MV (anterior leaflet)	1.2 × 1.0 × 0.5	Light-yellow neoplasms resembling sea anemones with looking like fronds surface Fig. 4a	Papillary fibroelastoma Fig. 4b	Complete resection of neoplasm and leaflet, valve replacement with 27-mm Edwards bioprosthesis valve	6.5 Normal bioprosthesis valve function, NYHAI
7	10	M	10	MV (anterior and posterior leaflet)	Multiple 0.2 × 0.1 × 0.1 0.4 × 0.2 × 0.1	White neoplasms looked like granular surface Fig. 3a	Myxoma Fig. 3b	Complete resection of neoplasm and leaflet, valve replacement with 25-mm SJM mechanical valve	4.5 Normal mechanical valve function, Without anticoagulation comlications INR 2.0 ~ 2.5, NYHAI
8	62	M	2	PV	10 × 5.5 × 4.5	Giant light-yellow neoplasms with complete capsule, destroyed the original PV Fig. 5a	Lipoma Fig. 5b	Complete resection of neoplasm and leaflet, valve replacement with 23-mm Edwards bioprosthesis valve	1 Normal bioprosthesis valve function, NYHAII

*MV* mitral valve, *AV* aortic valve, *TV* tricuspid valve, *PV* pulmonary valve

## Results

There were no complications and hospital deaths in the group. Follow-ups were mean 7.06 ± 4.24 (1–16) years. The malignant neoplasm patient underwent three cycles of intravenous systemic chemotherapy in 6 months postoperatively. All patients recovered a good left ventricular function with an ejection fraction of 67.38 ± 5.42 (58–74) percent. 5 patients (62.5 %) were asymptomatic. Heart functions recovered to New York Heart Association (NYHA) classIin 5 patients (62.5 %) and class II in 3 patients (37.5 %). The case 5 and 7 of who were replaced with mechanical valves took orally warfarin anticoagulation therapy and maintained INR 2.0–2.5, no blood-clotting and bleeding complications. The old man (case 2) with aortic valve tumor and associated with coronary artery diease took Lotensin (benazepril hydrochloride), Zebeta for the long-standing hypertension and Aspirin, Plavix for the antiplatelet therapy. Echocardiography showed postoperatively normal prosthetic valve function and without progressing mild AV regurgitation in 1 patient (12.5 %).

The maximum diameter of tumors were 2.80 ± 3.19 (0.4–10) cm. Pathology analysis revealed benign tumors in 7 patients (87.5 %) and malignant in 1patient (12.5 %). Myxoma was the most common classification of primary cardiac valve tumor. There were 3 myxoma patients in the group (37.5 %). Case 7 was diagnosed as a multiple myxoma on the mitral valve. Papillary fibroelastoma was the second common type (25 %). There were 1 rhabdomyoma (12.5 %), 1 lipoma (12.5 %) and 1 mesenchymal sarcoma (12.5 %) in the serie. The Mitral valve was the most primary located valve. There were 5 patients with primary mitral valve tumors (62.5 %). The mesenchymal sarcoma on posterior leaflet of mitral valve was the only malignant tumor (12.5 %). Four of mitral valve benign tumors were classified myxoma in 2 cases (25 %), including 1 multiple myxoma, rhabdomyoma in 1 case (12.5 %) and papillary fibroelastom in 1 case (12.5 %). The tricuspid valve tumor was a benign myxoma (12.5 %). The aortic valve tumor was a papillary fibroelastom (12.5 %). The neoplasma on pulmonic valve was the biggest one in size, and was a lipoma (12.5 %).

### Comment

Primary cardiac valve tumors are quite rare, and most of the detail information was obtained from autopsy [1, 2]. From January 1980 to December 2014, a total of 211 cases of surgical treatment of primary cardiac tumors were recorded in all of 28, 250 cases of intracardiac procedures, which occurred only 8 cases (3.8 % and 0.00028 %) with primary cardiac valve tumors in our department of cardiovascular surgery. According to reports in literatures, lots of valve tumors were asymptomatic, especially at the early stage. Before decades, the understanding of primary cardiac valve tumors had been derived from some autopsy findings and limited case reports, but as more and more surgeons found in clinical work and study, also with the progress of modern medical diagnostic technologies, primary cardiac valve tumors were gradually becoming less rare in clinic. This has been most directly attributable to the technologic advancements and more frequent use of echocardiography.

### Clinical features and diagnoses

The clinical presentations were determined by many factors, including tumor classifications, location, size, growth rate, and tendency to embolization and so on. The classical presentations of cardiac tumor clinical are the congestive heart failure caused by intracardiac obstruction, signs of embolization, constitutional symptoms of fever and weight loss or fatigue, and immunologic manifestations of myalgia, weakness, and arthralgia, patients presenting with one or more of these symptoms. Symptoms are usually due to obstructions, compression of cardiac cavities and valvular malfunction. Cardiac rhythm disturbance and infection occur less frequently. Congestive heart failure could be elicited by tumor-induced valve dysfunction, as well as tumor-related serious obstruction of blood flow across the valve. Cerebral, coronary, pulmonic, and retinal embolisms have been reported in many primary valve tumor patients. The emboli might come from either neoplasm fragments or thrombus around the neoplasm. Mitral valve tumor was most frequently associated with embolism [2, 3]. Two patients (25 %) with a cerebral embolism were proved to be aortic and mitral valve papillary fibroelastoma in the group. Pathologically, papillary fibroelastoma and myxoma are the most likely to be related to embolism [2, 3]. In the group, the congestive heart failure resulting from tumor-induced valve dysfunction was found in 3 patients (37.5 %). Systemic symptoms such as weight loss, fever, and arthralgia were not typical symptom. There were some obvious systemic symptoms whether benign or malignant neoplasm in lots of patients. In the past, it is difficult to diagnosis in early stage of the disease without specific clinical presentation. With the development of technology, especially with echocardiography be used widespreadly, to diagnose asymptomatic patients become easier. There were some other methods, such as computed tomography and magnetic resonance imaging to be used in clinic. However, there is no definitive feature of distinguishes types of intracardiac valve masses. Thrombus, bacteria vegetations, calcifications are some tumor-like lesions on cardiac valves. They may have similar morphological and clinical characteristics, round appearance, small size, high mobility with movement of the valves, predisposition for embolic events, and amenable to surgical resection.

### Classifications of pathology and locations

In papillary fibroelastoma cases, as one of the most common types of primary valve tumor, echocardiography would provide satisfactory information for diagnoses. Grossly, they have been compared to a sea anemone because of their numerous and delicate papillary fronds. Sun and colleagues [4, 5] summarized the typical echocardiographic features of these particular tumors: round, oval, or irregular in shape with clear borders and homogeneous textures, small in size in most instances, pedunculate and mobile in nearly more than half of cases, and arising from the aortic valve in the majority of cases and from the mitral valve secondly. It represents the most common tumor of the heart valves and accounts for 7.9 % of benign primary cardiac tumors. The aortic valve is the most (44 %) followed by the mitral valve (35 %), the tricuspid valve (15 %) and the pulmonary valve (8 %). Other sites involved were the left atrium, atrial septum, right atrium, atrioventricular valve, right ventricle, pulmonary valve, mitral chorda, and left ventricular apex [6]. Cytomegalovirus has been recovered in these tumors suggesting the possibility of viral induction of the tumor and chronic viral endocarditis [7]. The majority of papillary fibroelastomas presents as solitary masses, but 5.2 % presents as multiple tumors up to 8 tumors at various locations in the heart [6]. They are usually asymptomatic until a critical event occurs. Patient 6, a 26-year-old lady, was admitted to local hospital with complaints of speech slowly and limb weakness that developed over the last 6 h. Subsequent cranial computed tomography scan demonstrated an infarct zone in the right centrum semiovale. Echocardiography revealed a mobile, rounded echogenic mass attached to the anterior leaflet of mitral valve. Prompt surgical resection is necessary to prevent potential embolic and other critical events.

Myxoma occurs in any chamber of the heart but have a special predilection for the left atrium, from which approximately 75 % originated [8]. Myxoma is the other most common type of benign neoplasm in cardiac valve [9, 10]. There were 3 patients with valvular myxoma (case 1,3 and 7), including a 10-year-old boy with a multiple myxoma on the mitral leaflets (case 7) and an adult with myxoma on the tricuspid valve (case 3) in the series.

Rhabdomyoma is the most frequently occurring cardiac tumor in child [11, 12]. A one-year-old boy with rhabdomyoma on the mitral anterior leaflet was the youngest patient in the series (case 4). A peculiar feature of cardiac rhabdomyoma is its spontaneous regression, particularly of smaller lesions, followed by a resolution of symptoms. Over 90 % of rhabdomyomas are multiple and occur with approximate equal frequencies in both ventricles [12]. It is thought to be a myocardial hamartoma rather than a true neoplasm. Fifty percent of patients with tuberous sclerosis have rhabdomyoma but more than 50 % of patients with rhabdomyoma have or will develop tuberous sclerosis [13, 14].

Pulmonary valve lipoma is extremely rare [15]. It rarely obstructs blood flow, since cardiac lipoma is usually intramural or epicardia in location. Large subpericardial lipoma exerting pressure on cardiac structures may determine angina or interfere with pump function; intramyocardial lipomas can produce damages to normal electric conduction and arrhythmias [16]. The giant lipoma (case 8) can cause either severe valve regurgitation or mechanical obstruction. The congestive heart failure is due to the pulmonary valve dysfunction and right ventricular outflow tract obstruction.

Approximately 25 % of primary cardiac tumors are malignant (and of these), about 75 % of which are sarcomas [17]. If the complete resection is possible, surgery provides better palliation and can possibly double survival [18]. A complete resection depends on the location of the tumor, extent of involvement of the myocardium and/or fibrous skeleton of the heart, and histology.

### Treatments and prognosis

Therapeutic aims are total tumor resection and restoration of cardiac valve function. Surgical treatment should not be delayed because death from obstruction or embolization may occur in as many as 8 % of patients awaiting operations [19, 20]. Median sternotomy approach with ascending aortic and bicaval cannulation are usually employed. The Surgical resection using cardiopulmonary bypass with cardioplegic arrest is the best method for patients with cardiac valve tumors whether benign or malignant neoplasm. The valve reconstruction avoids prosthesis-related complications, such as heart block, paravascular leak, or the need for anticoagulation. If malignancy is suspected or confirmed, and if the lesion appears anatomically resectable and there is no metastatic disease, then resection should be considered. Fortunately, the only malignant patient (case 5) was completely excised tumor, mitral valve and subvalvular apparatus. She was underwent intravenous systemic chemotherapy for three intravenous systemic chemotherapy cycles in 6 months postoperatively. We recommend adjuvant chemotherapy and believe this will slightly improve survival [17, 18]. Following the tumor removal from the field, the area should be liberally irrigated, suctioned, and inspected for loose fragments. Sometimes, it is still difficult to determine whether the tumor is benign or malignant only by its gross appearance. Therefore, frozen sections of the tumor must be obtained for differentiation in any questionable case. The pathological examination is very important. It guide treatment strategies during and post operations. According to reports [19, 20], the prognosis for patients with a malignant valve tumor was poor. Early diagnoses, complete tumor resections, prosthetic valve replacements and affected adjuvant therapies are key strategies to the valve tumor patients. The tumor is too small to be seen at routine echocardiography, interferences caused by combined lesions and lack of sufficient knowledge are main reasons for missing and misdiagnosis of the primary valve tumor in the early stage. The diagnoses are based on imaging findings and the classical triad symptoms associated with the hemodynamic abnormalities, the organ embolism and the systemic symptoms directly from tumors. Early diagnosis and intervention are keys to reserve the normal original valve function. Prompt surgical resection is necessary to prevent potential critical events.
